# Genetically predicted circulating levels of cytokines and the risk of oral cavity and pharyngeal cancer: a bidirectional mendelian-randomization study

**DOI:** 10.3389/fgene.2023.1321484

**Published:** 2024-01-11

**Authors:** Kehan Wu, Qianhui Sun, Dongxu Liu, Jiayi Lu, Deyu Wen, Xiyan Zang, Li Gao

**Affiliations:** ^1^ Department of Oral and Maxillofacial Surgery, The Second Affiliated Hospital of Harbin Medical University, Harbin, Heilongjiang, China; ^2^ Department of Cardiology, The Second Affiliated Hospital of Harbin Medical University, Harbin, Heilongjiang, China

**Keywords:** oral cavity and pharyngeal cancer, inflammation factors, cytokines, mendelian randomization, HNSCC, genome-wide association study

## Abstract

**Background:** Epidemiological research has established associations between various inflammatory cytokines and the occurrence of oral cancer and oropharyngeal cancer (OCPC). We performed a Mendelian randomization (MR) analysis to systematically investigate the causal relationship between inflammatory cytokines and OCPC.

**Methods:** We performed a bidirectional two-sample MR analysis using OCPC from 12 studies (6,034 cases and 6,585 controls) and genome-wide association study (GWAS) results for 41 serum cytokines from 8,293 Finns, respectively. Inverse variance weighting was used as the primary MR method and four additional MR methods (MR Egger, Weighted median, Simple mode, Weighted mode) were used to examine genetic associations between inflammatory traits and OCPC, and Cochran’s Q test, MR-Egger intercept, leave-one-out analysis, funnel plot, and multivariate MR (MVMR) analysis were used to assess the MR results.

**Results:** The results suggested a potential association between high gene expression of Macrophage inflammatory protein-1α (MIP1α/CCL3) and an increased risk of OCPC (Odds Ratio (OR): 1.71, 95% Confidence Interval (CI): 1.09–2.68, *p* = 0.019). Increasing the expression levels of the interleukin-7 (IL-7) gene by 1 standard deviation reduced the risk of OCPC (OR: 0.64, 95%CI: 0.48–0.86, *p* = 0.003). In addition, multivariate Mendelian randomization analysis also showed the same results (MIP1α/CCL3, OR: 1.002, 95% CI: 0.919–1.092, *p* = 0.044; IL-7, OR: 0.997, 95% CI: 0.994–0.999, *p* = 0.011). Conversely, there was a positive correlation between genetic susceptibility to OCPC and an increase in Interleukin-4 (IL-4) (OR: 1.04, 95%CI: 1.00–1.08, *p* = 0.027).

**Conclusion:** Our study systematically assessed the association between inflammatory cytokines and the risk of OCPC. We identified two upstream regulatory factors (IL-7 and CCL3) and one downstream effector factor (IL-4) that were associated with OCPC, offering potential avenues for the development of novel treatments.

## 1 Introduction

Based on the latest estimates from Global Cancer Statistics 2020, Head and Neck Squamous Cell Carcinoma (HNSCC) ranks as the seventh most prevalent cancer worldwide, with an annual incidence of over 890,000 new cases and a mortality of over 450,000 (Sung et al., 2021). Among HNSCC cases, Oral Squamous Cell Carcinoma (OSCC) and Oropharyngeal Squamous Cell Carcinoma (OPSCC) are predominant, contributing to a global incidence of over 260,000 cases and over 128,000 deaths, respectively (Siegel et al., 2020). Despite some advances in the treatment of oral and oropharyngeal cancer (OCPC), the 10-year survival rate remains low at below 60%. Even if the treatment is successful, patients may still experience severe functional impairments, including compromised abilities in feeding, swallowing, and speech. Additionally, the recurrence rate remains high (Sung et al., 2021).

Earlier investigations in preclinical settings have indicated that inflammatory cytokines, such as TNF-α, IL-1*β*, and IL-6, promote the growth, invasion, and spread of cancer cells. Additionally, the transcription factors associated with these cytokines such as NF-kB and STAT3 show increased expression in most cancer types (Voronov et al., 2003; Pikarsky et al., 2004; Bierie and Moses, 2006; Luo et al., 2007). For example, inhibiting the activity or expression of IL-1*β* can prevent the occurrence of oral cancer by regulating specific key node genes in the tumor microenvironment (TME) (Wu et al., 2016). Moreover, mounting evidence indicates an increased risk of OCPC in the presence of inflammation, and inflammation commonly accompanies the development of OCPC (Leon et al., 2015). These observations suggest that the pharmacological targeting of additional inflammation biomarkers identified in the epidemiological literature through observational studies could offer a potentially effective approach for treating OCPC (Todoric et al., 2016). Nevertheless, the investigations conducted thus far have primarily concentrated on a limited range of inflammatory elements and have failed to acknowledge the impact of additional factors on the changes in inflammation levels. Hence, it is crucial to ascertain whether the changes in inflammatory factors cause the onset of tumors or if the tumors themselves modify the microenvironment, leading to differences in inflammatory factors. Due to the limited comprehension of the cause of OCPC, investigating the exact characteristics of the connections between inflammatory factors and OCPC has considerable clinical significance.

The dynamic nature of the inflammatory response suggests that a specific time point’s measurement, whether high or low, might not precisely represent the overall trend of inflammatory factor variations. Epidemiological, genetic, and biological investigations have confirmed the link between inflammatory factors and OCPC. However, the outcomes derived from these investigations may be distorted by unforeseen confounding variables or reverse causal associations, thus complicating the establishment of clear causal relationships.

Observational studies may hinder an exhaustive comprehension of the connection between OCPC and inflammation due to their intrinsic research limitations. Thus, a detailed understanding of the role of circulating cytokines and their association with OCPC risk may aid in the development of prevention, prediction, and treatment strategies. Researchers may achieve a more comprehensive understanding of the connection between inflammation and OCPC by addressing these limitations.

Mendelian randomization (MR) is a commonly used tool in genetic epidemiology (10), which utilizes instrumental variable IV) variation derived from non-experimental data to assess the causal impact of exposure (e.g., circulating cytokines) on an outcome (e.g., OCPC) (Lawlor et al., 2008). Given the random allocation of alleles during meiosis, MR can mitigate confounding variables and reverse causality, thereby offering more robust evidence for causal inferences (Burgess et al., 2015). The use of two-sample MR analysis allows researchers to evaluate the associations between the instrument-exposure and instrument-outcome in two distinct population samples, thereby improving the applicability and efficiency of testing (Hartwig et al., 2016). In this study, we performed an analysis of the genome-wide association study (GWAS) summary data of 41 inflammatory cytokines to identify relevant genetic variations. These variations were then further investigated in relation to OCPC. Specifically, we examined the correlations between these genetic variations and OCPC by reversing the exposure and outcome. Our research results not only provided substantial Supplementary Data for previous epidemiological investigations but also offered fresh perspectives on the development and prevention of OCPC.

## 2 Methods

### 2.1 Study design

**FIGURE 1:**
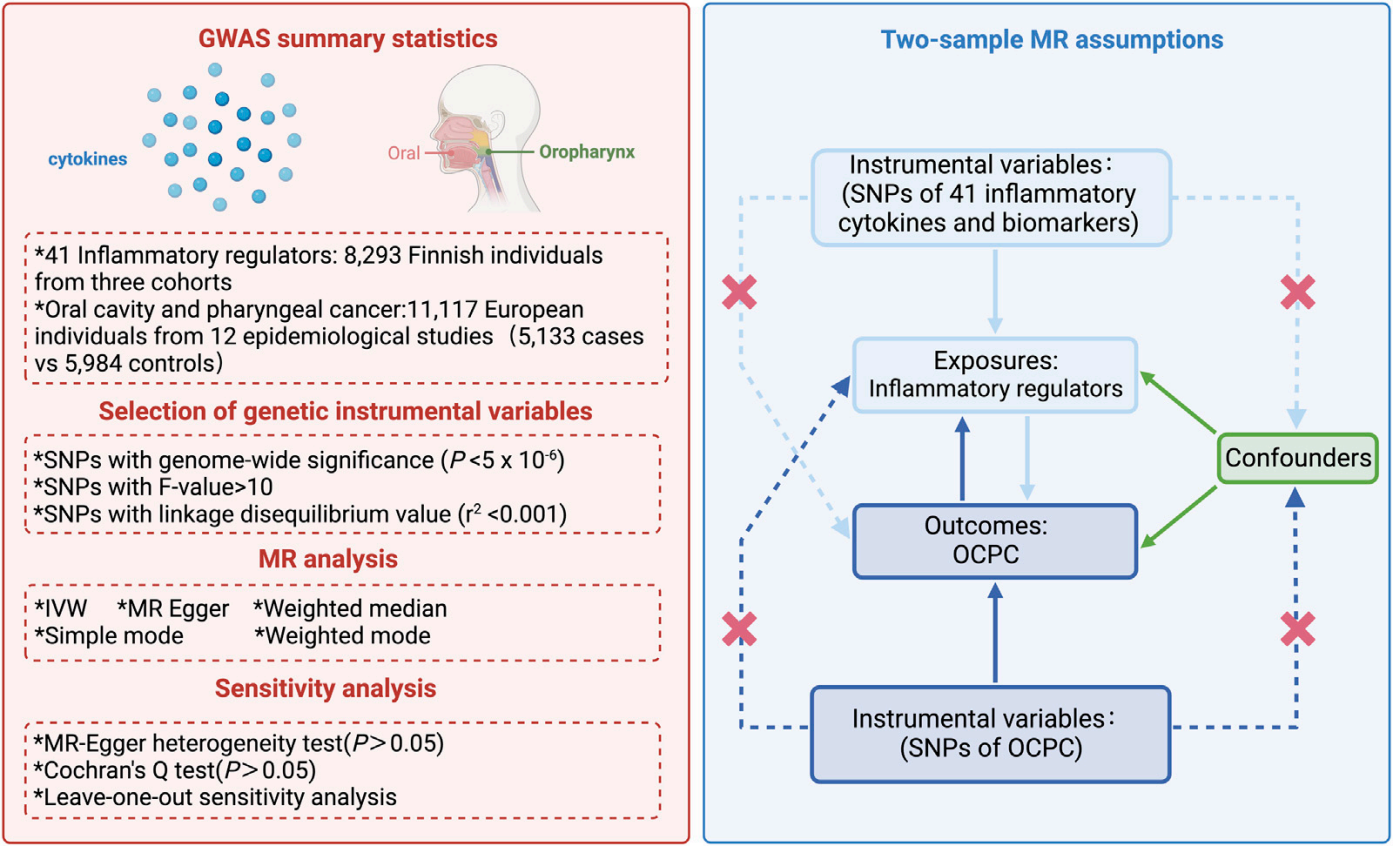
Illustration of the research design in the bidirectional Mendelian randomization (MR) analysis. Important instrumental variables, including 41 inflammatory cytokines and OCPC, were selected to explore the bidirectional causal relationships. This method is used to detect correlations (solid line) and violations of the Mendelian randomization assumption (dashed line). This figure was created using BioRender.com.

This study used a bidirectional MR approach to assess the causal relationship between the circulating cytokines and OCPCs. The study’s overall design is illustrated in Figure 1. To assess the causal relationship between circulating cytokines and OCPCs, MR analysis was performed to test the following three hypotheses: 1) Genetic instruments have a strong association with exposure; 2) Genetic instruments are not affected by any potential known confounding factors; 3) The association between the genetic instrument and the outcome is solely influenced by the exposure (Smith and Ebrahim, 2003). Simultaneously, the reverse MR method is used to examine potential reverse causal effects. The data used in this study were obtained from publicly available large-scale GWAS which can be accessed through the GWAS Catalog service (https://www.ebi.ac.uk/gwas/home). The studies included in the initial GWAS had obtained approval from their respective institutional review boards.

### 2.2 Genetic instrumental variables for inflammatory factors

The research used an extensive meta-analysis of GWAS on the circulating levels of 41 cytokines. This analysis combined information from three separate cohort studies: FINRISK 1997, FINRISK 2002, and the Young Finns Study on Cardiovascular Risk (YFS). This study included a total of 8,293 participants. The genomic and cytokine data were contributed by 4,608 individuals from FINRISK 1997, while another 1,705 participants from FINRISK2002 also provided their cytokine data. Cytokine quantification was performed by analyzing the EDTA-treated plasma of FINRISK 1997, the heparinized plasma of FINRISK 2002, and the serum samples from YFS. To control for potential confounding factors, such as age, sex, body mass index, and genetic variations, genetic associations were properly adjusted. Specifically, the top 10 genetic principal components were included using genomic control correction. Bio-Rad’s pre-mixed Bio-Plex Pro Human Cytokine 27-plex Assay and 21-plex Assay were used to measure a total of 41 cytokines in YFS and FINRISK 2002, respectively. The measurements were performed by the Bio-Plex 200 reader equipped with Bio-Plex 6.0 software. However, it is important to note that, out of the 41 cytokines, 7 had to be excluded due to the presence of missing values exceeding 90% (Ahola-Olli et al., 2017; Kalaoja et al., 2021). A detailed overview of the GWAS data for the cytokines used in the MR analysis is presented in Table 1.

**TABLE 1:** Sample size for each cytokine analyzed in this study acquired from the GWAS.

Cytokines		Abbreviation	Sample size	Number
Chemokines	Cutaneous T cell attracting (CCL27)	CTACK	3,631	GCST004420
	Eotaxin (CCL11)	Eotaxin	8,153	GCST004460
	Growth regulated oncogene-α (CXCL1)	GROα	3,505	GCST004457
	Interferon gamma-induced protein 10 (CXCL10)	IP10	3,685	GCST004440
	Monocyte chemotactic protein-1 (CCL2)	MCP1	8,293	GCST004438
	Monocyte specific chemokine 3 (CCL7)	MCP3	843	GCST004437
	Monokine induced by interferon-gamma (CXCL9)	MIG	3,685	GCST004435
	Macrophage inflammatory protein-1α (CCL3)	MIP1α	3,522	GCST004434
	Macrophage inflammatory protein-1*β* (CCL4)	MIP1*β*	8,243	GCST004433
	Regulated on Activation, Normal T Cell Expressed and Secreted (CCL5)	RANTES	3,421	GCST004431
	Stromal cell-derived factor-1 alpha (CXCL12)	SDF1α	5,998	GCST004427
Growth factors	Beta nerve growth factor	*β*NGF	3,531	GCST004421
	Basic fibroblast growth factor	bFGF	7,565	GCST004459
	Granulocyte colony-stimulating factor	GCSF	7,904	GCST004458
	Hepatocyte growth factor	HGF	8,292	GCST004449
	Macrophage colony-stimulating factor	MCSF	840	GCST004436
	Platelet-derived growth factor BB	PDGFbb	8,293	GCST004432
	Stem cell factor	SCF	8,290	GCST004429
	Stem cell growth factor beta	SCGF*β*	3,682	GCST004428
	Vascular endothelial growth factor	VEGF	7,118	GCST004422
Interleukins	Interleukin-10	IL-10	7,681	GCST004444
	Interleukin-12p70	IL-12p70	8,270	GCST004439
	Interleukin-13	IL-13	3,557	GCST004443
	Interleukin-16	IL-16	3,483	GCST004430
	Interleukin-17	IL-17	7,760	GCST004442
	Interleukin-18	IL-18	3,636	GCST004441
	Interleukin-1 receptor antagonist	IL1ra	3,638	GCST004447
	Interleukin-1-beta	IL-1*β*	3,309	GCST004448
	Interleukin-2	IL-2	3,475	GCST004455
	Interleukin-2 receptor, alpha subunit	IL2rα	3,677	GCST004454
	Interleukin-4	IL-4	8,124	GCST004453
	Interleukin-5	IL-5	3,364	GCST004452
	Interleukin-6	IL-6	8,189	GCST004446
	Interleukin-7	IL-7	3,409	GCST004451
	Interleukin-8 (CXCL8)	IL-8	3,526	GCST004445
	Interleukin-9	IL-9	3,634	GCST004450
Others	Interferon-gamma	IFN-γ	7,701	GCST004456
	Macrophage migration inhibitory factor (glycosylation-inhibiting factor)	MIF	3,494	GCST004423
	Tumor necrosis factor-alpha	TNFα	3,454	GCST004426
	Tumor necrosis factor-beta	TNF*β*	1,559	GCST004425
	TNF-related apoptosis inducing ligand	TRAIL	8,186	GCST004424

### 2.3 Genetic instrumental variables for OCPC

Using data from the most extensive GWAS to date, we investigated the impact of inflammatory elements on the vulnerability to OCPC. By extracting specific variations in single-nucleotide polymorphisms (SNPs) linked to exposure, we assessed a total of 6,034 cases and 6,585 controls enrolled in 12 different epidemiological studies (Lesseur et al., 2016). The Genetic Associations and Mechanisms of Oncology (GAME-ON) network is responsible for conducting these studies. Every participant has provided informed consent, and the relevant institutional review boards have approved this study. Furthermore, the study includes data from the European Prospective Investigation into Cancer and Nutrition (EPIC) study, and the Health and Nutrition 5,000 (HN5000) study, which specifically examines the health and lifestyle of 5,000 United Kingdom participants. Extensive information about the study, as well as the genotyping and imputation methods used, has been previously documented (Lesseur et al., 2016). The study sample comprised individuals from Europe (45.3%), North America (43.9%), and South America (10.8%). The study encompasses diverse types of cancer identified through the specific International Classification of Diseases, 10th Revision (ICD-10) codes, such as oral cancer (C02.0–C02.9, C03.0–C03.9, C04.0–C04.9, C05.0–C06.9) and oropharyngeal cancer (C01.9, C02.4, C09.0–C10.9). Exclusion criteria led to the removal of 954 individuals with hypopharyngeal cancer, unidentifiable codes, or overlapping cancers. To mitigate the effects of heterogeneity in these regions, GWAS studies were conducted exclusively on individuals with European ancestry, totaling 11,117 participants. Among these, there were 5,133 cases, comprising 2,700 cases of oral cancer and 2,433 cases of oropharyngeal cancer. In addition, there were 5,984 control cases (Supplementary Table S1).

### 2.4 Assessing common risk factors for OCPC

The cytokines-OCPC pathway is a potential target for cancer prevention and treatment. To identify the possible mediators of this pathway, we applied MR methods to examine the causal links between cytokines and common risk factors for OCPC. We used an existing database to investigate the following risk factors that have been widely recognized as associated with OCPC: smoking, drinking, body mass index, type 2 diabetes, hypertension, and HPV16/18 infection (Argiris et al., 2008; Leemans et al., 2018). Supplementary Table S2 lists the GWAS summary data for the above risk factors.

### 2.5 Selection of genetic instrumental variables

To ensure that the conclusion regarding the causal relationship between cytokines and the risk of OCPC is authentic and accurate, we implement various quality control measures in the selection of optimal genetic instrumental variables. Our approach involves the selection of SNPs that are closely associated with inflammatory factors and demonstrate genome-wide significance with a *p*-value lower than 5 × 10^−8^. By focusing on these specific SNPs, we establish robust instrumental variables for our study (Burgess et al., 2011). We proceeded to eliminate linkage disequilibrium (LD). Our criteria for removal included an *r* ^2^ value below 0.001 and a distance of 5,000 kb. SNPs exceeding the *r* ^2^ threshold of 0.001, which includes the most significant SNP within a 5,000 kb range, were excluded. After aligning the chosen SNPs with the outcome data, we found that only ten out of the 41 systemic inflammation factors available exhibited two or more independent SNPs at a significance level of *p*-value less than 5 × 10^−8^. Additionally, it was found that nine of them displayed three or more independent SNPs. Since inflammatory cytokines are a class of cytokines with multiple functions and interactions, and they may involve multiple genes and SNPs, a less stringent criterion is needed to capture their genetic variation. When selecting instrumental variables, we established A less stringent *p*-value threshold (5 × 10^−6^) to capture more SNPs of inflammatory cytokines (Li et al., 2021; Pan et al., 2023). If there is an insufficient number of exposure-associated SNPs detected in the GWAS findings, proxy SNPs exhibiting high LD with a correlation coefficient (*r* ^2^ > 0.90) will be employed as alternative substitutes. These proxy SNPs can be accessed through LDlink (https://ldlink.nci.nih.gov/) as a resource (Machiela and Chanock, 2015). To exclude all SNPs associated with exposure and avoid potential pleiotropic effects, we performed a comprehensive investigation using the PhenoScanner V2 tool (http://www.phenoscanner.medschl.cam.ac.uk/) (Staley et al., 2016). Through the aforementioned steps, we acquired 41 cytokines associated with inflammation. As an instrumental variable with a significance threshold of F < 10 is considered weak, we will exclude it from our study. Detailed information on the identified SNPs is listed in Supplementary Tables S3 and S4. Furthermore, to uphold the fundamentals of MR, we shall examine the desired SNPs to exclude any that exhibit associations with the resulting outcomes.

### 2.6 MR statistical analysis

To explore the causal relationship between inflammatory regulators and OCPC, we utilized GWAS data and employed two-sample MR and multivariate MR methods. Statistical analysis was conducted using R software (v4.1.3) and the “MendelianRandomization”, “MVMR” and “MRPRESSO” software packages. Multivariable MR (MVMR) is mainly used to evaluate the impact of multiple potential exposures on overall outcomes and identify potential risk factors (Tian and Burgess, 2023). The relationship between inflammatory factors and OCPC was examined using the inverse variance weighted (IVW) method. The instrumental variable method was used to estimate the mean effects of SNPs by regressing SNP-inflammatory factors on SNP-OCPC. Additionally, we utilized the weighted median estimator (WME) and the MR-Egger regression. WME is a statistical method that applies weights to the empirical distribution function of ratio estimates for SNPs within the study range, minimizing biases in estimating causal effects. MR-Egger regression employs weighted linear regression to estimate the effect of SNP-OCPC, considering SNP-inflammatory factors, and provides an evaluation of causal effects, even in the presence of invalid instruments (Bowden et al., 2015). If the directions of the *β*-values of other methods are the same, it may be interpreted as a positive result (Chen et al., 2020). Moreover, the “Leave-one-out” strategy was employed to visually illustrate whether a single SNP substantially influences the causal association. To ensure the dependability of the MR findings, we performed diverse evaluations of diversity and sensitivity. We employed Cochran’s Q test to assess heterogeneity among SNPs. If no indications of heterogeneity were observed, we utilized a fixed-effect model; otherwise, a random-effects model was implemented. The Egger-intercept test was carried out for horizontal pleiotropy examination (Verbanck et al., 2018; Song et al., 2022). The results of the study were presented using odds ratios (ORs) and their corresponding 95% confidence intervals (CIs). Any results with a *p*-value less than 0.05 were considered statistically significant. We applied the FDR method to correct for multiple testing, which is a method implemented by the *q*-value program. It is a commonly used method for multiple testing correction, which can control the proportion of false rejections of the null hypothesis among multiple hypothesis tests. We chose a *q*-value of less than 0.1 as the significance level, which is a reasonable choice because it can reduce the false positive rate while maintaining the statistical power. When the *p*-value is less than 0.05 but the *q*-value is greater than or equal to 0.1, we consider it a suggestive association result (Storey and Tibshirani, 2003; Li et al., 2022). The analysis of the MR study adhered to the guidelines set forth by the Strengthening the Reporting of Observational Studies in Epidemiology using Mendelian Randomization (STROBE-MR) statement, which emphasizes the scientific rigor and reporting standards for observational studies in epidemiology (Skrivankova et al., 2021).

## 3 Results

### 3.1 Causal impact of systemic inflammation factors on the risk of OCPC

**FIGURE 2:**
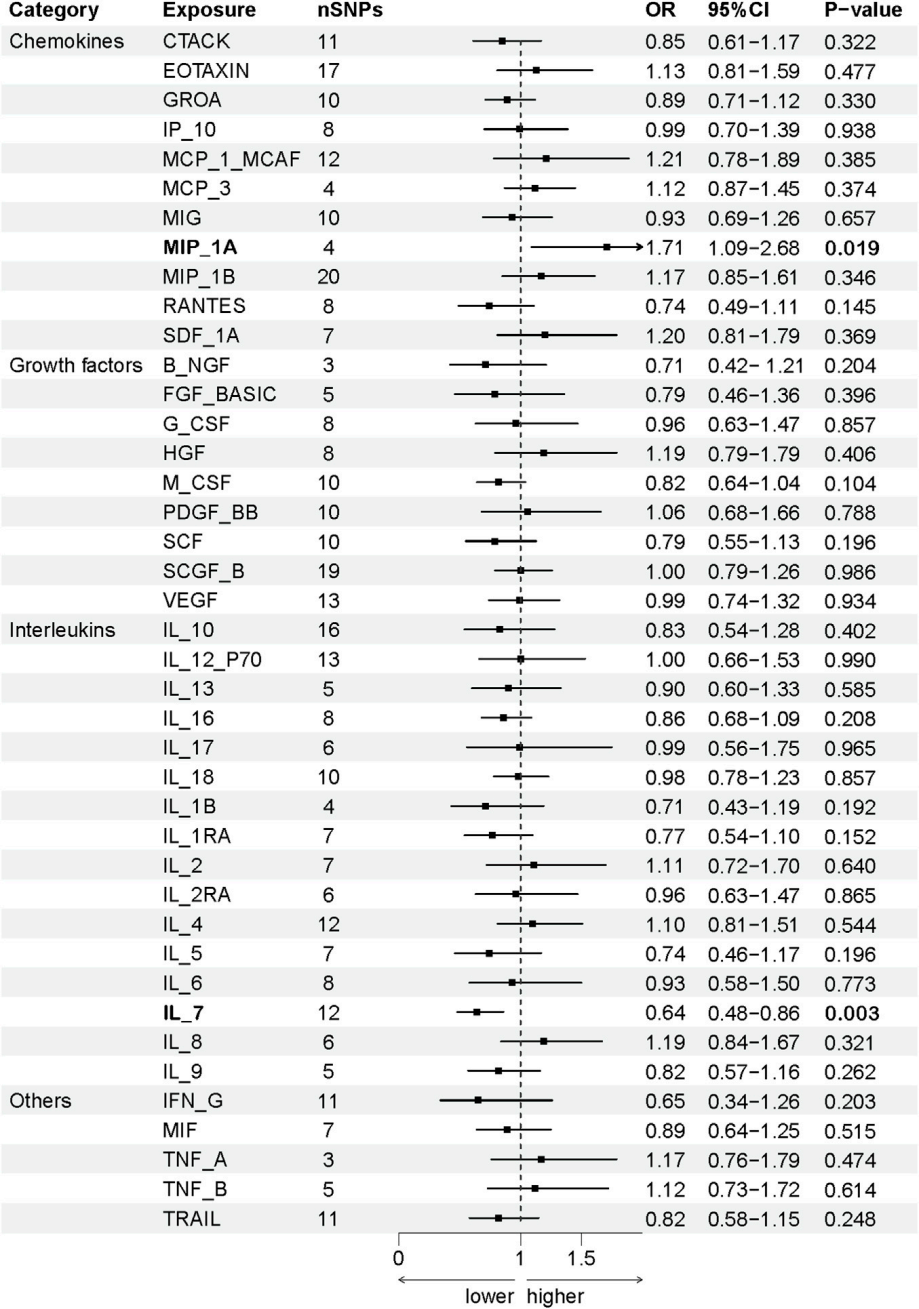
Using SNPs with a significance level of *p* < 5 × 10^−6^, we predicted the potential impact of inflammation regulatory factors across the genome on OCPC. To establish a causal link between circulating cytokine levels and OCPC, we conducted a two-sample Mendelian randomization (MR) analysis employing the IVW method. By estimating the odds ratio of OCPC for every 1-SD rise in predicted circulating cytokine levels, as determined by genetic prediction, we derived a 95% confidence interval (CI) value. This approach primarily determines the causal relationship between OCPC and the levels of circulating cytokines. A detailed overview of cytokines is provided in Table 1.

**FIGURE 3:**
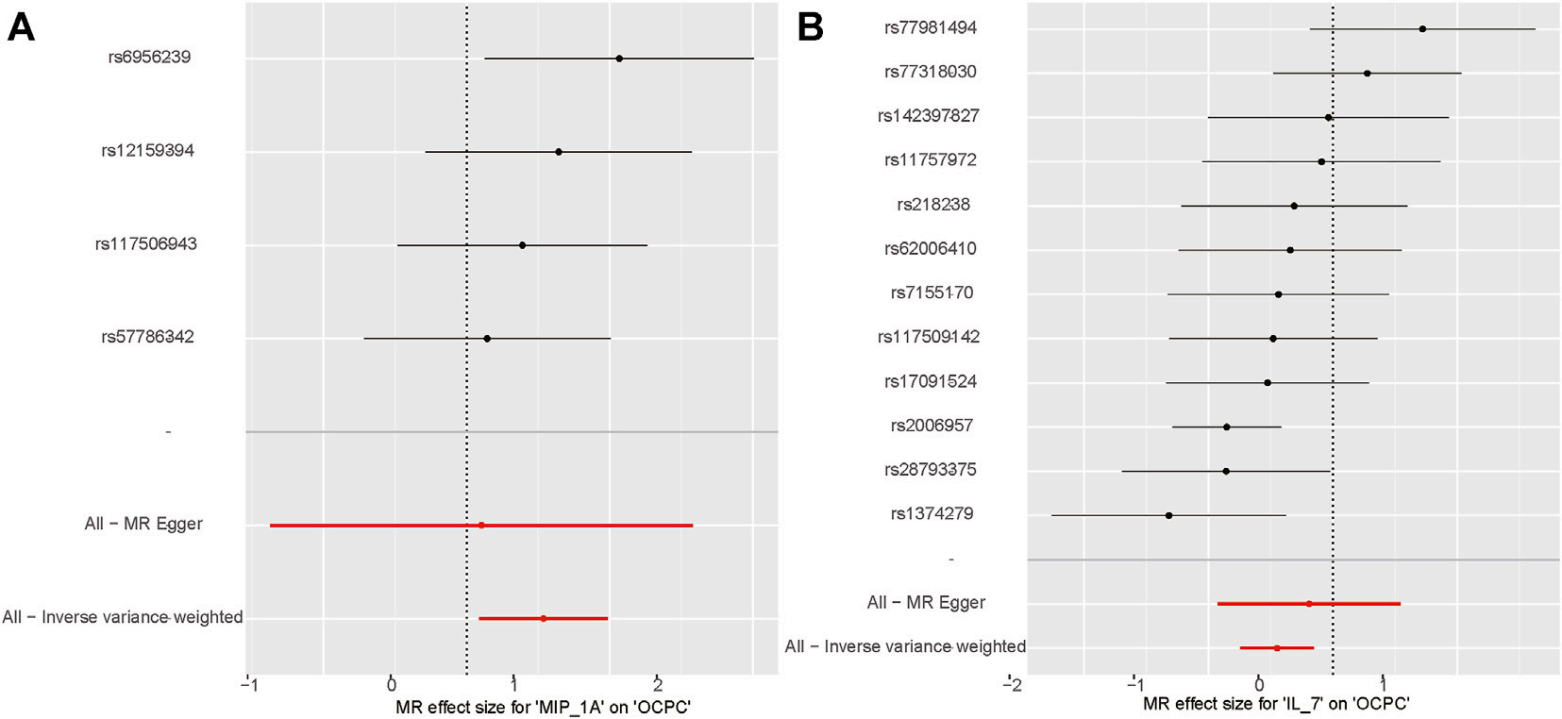
MIP1α/CCL3 and IL-7 related SNPs are depicted in the forest plot for OCPC risk. **(A)** Forest plot for MIP1α/CCL3 exposure. **(B)** Forest plot for IL-7 exposure. The *X*-axis represents the effect estimates of MIP1α/CCL3 and IL-7 on OCPC analyzed using MR. The *Y*-axis represents the MR effect values for each SNP site.

**FIGURE 4:**
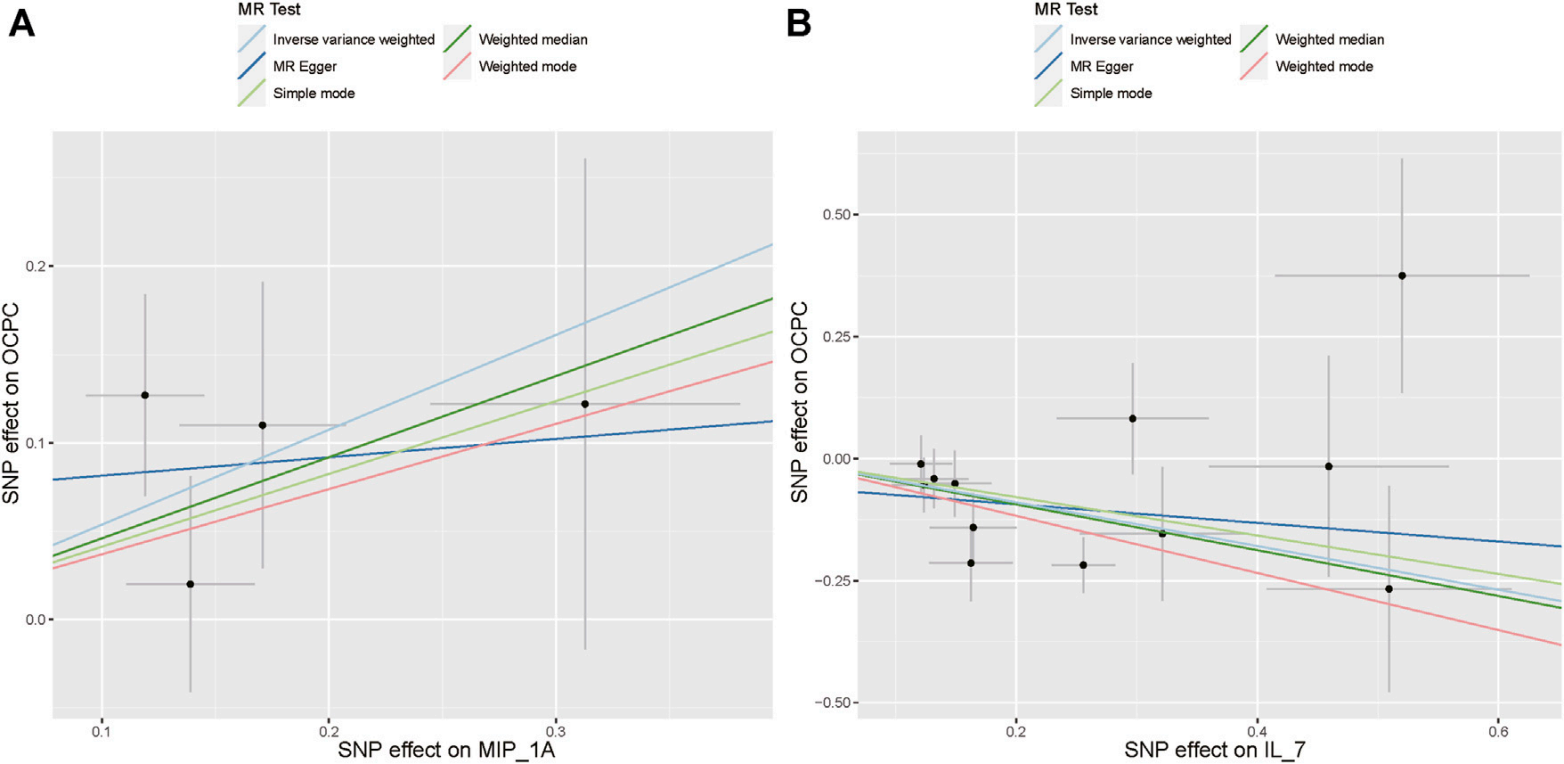
Using various MR methods, the scatter plots demonstrate the genetic correlation between SNP and OCPC for MIP1α/CCL3 and IL-7. **(A)** The exposure of MIP1α/CCL3 is illustrated in the scatter plot. **(B)** The exposure of IL-7 is illustrated in the scatter plot. Each scatter plot illustrates the associations between the alleles and the risk of the outcome, plotted against the association with one standard deviation of exposure. The effects are represented by the gray error bars, which indicate the 95% confidence intervals. The IVW, MR Egger, Weighted median, Simple mode, and Weighted mode were employed for the analysis. The estimated MR effect of each method can be determined by the slope of each line.

The association between systemic inflammation factors and OCPC was revealed through gene prediction, supported by the following findings (Figure 2). The IVW method uncovered a significant surge in OCPC risk, linked to higher levels of Macrophage inflammatory protein-1α (MIP1α/CCL3) (OR: 1.71, 95% CI: 1.09–2.68, *p* = 0.019). Both the MR-Egger heterogeneity test and Cochran’s Q test failed to identify any signs of heterogeneity, indicating an absence of variation (*p* > 0.05). Additionally, by utilizing the IVW method, increased Interleukin-7 (IL-7) levels were associated with a decreased likelihood of OCPC(OR: 0.64, 95%CI: 0.48–0.86, *p* = 0.003). No evidence of heterogeneity or horizontal pleiotropy was discovered (*p* > 0.05) (Table 2). Figures 3, 4 show forest plots and scatter plots illustrating the genetic association of MIP1α/CCL3 and IL-7 SNPs with OCPC. The funnel plot demonstrates overall symmetry, indicating little evidence of heterogeneity (Supplementary Figure S1). A sensitivity analysis, using a leave-one-out approach, provided evidence of the lack of a single SNP with substantial impact on the overall effect. This verifies the dependability and consistency of the estimation of the causal effect (Supplementary Figure S3).

**TABLE 2:** Our research examined whether there is a causal relationship between the levels of MIP1α/CCL3 and IL-7 in the circulatory system and OCPC. To achieve this, we employed genetic prediction techniques. To assess the variation in estimates of individual SNP effects, we used Cochran’s Q test, and we employed the MR-Egger intercept test to evaluate horizontal pleiotropy.

Cytokines	Methods	MR results					Heterogeneity test			Horizontal pleiotropy test		
							Cochran’s Q test			MR-egger intercept test		
		SNPs	*β*	SE	*P*	OR (95% CI)	Q	df	*P*	Intercept	SE	*P*
MIP1α/CCL3	IVW	4	0.537	0.229	0.019	1.711 (1.092–2.680)	2.188	3	0.534	0.071	0.118	0.607
	MR Egger	4	0.104	0.753	0.903	1.111 (0.254–4.852)	1.824	2	0.402			
	Weighted median	4	0.459	0.268	0.086	1.583 (0.937–2.674)						
	Simple mode	4	0.412	0.416	0.395	1.510 (0.668–3.412)						
	Weighted mode	4	0.369	0.378	0.390	1.446 (0.703–2.975)						
IL-7	IVW	12	−0.448	0.151	0.003	0.639 (0.475–0.859)	18.890	11	0.063	−0.055	0.073	0.468
	MR Egger	12	−0.191	0.374	0.621	0.826 (0.397–1.720)	17.875	10	0.057			
	Weighted median	12	−0.469	0.168	0.005	0.626 (0.450–0.870)						
	Simple mode	12	−0.394	0.319	0.242	0.674 (0.361–1.260)						
	Weighted mode	12	−0.586	0.246	0.036	0.557 (0.344–0.902)						

Abbreviations: SNP, single nucleotide polymorphism; *β*, effect size of SNP on exposure; SE, standard error; OR, odds ratio; CI, confidence interval; df, degrees of freedom; IVW, inverse variance weighted.

Supplementary Table S5 presents the MR outcomes concerning the prediction of genetic susceptibility to systemic inflammation factors and OCPC risk assessment. Furthermore, it contains summaries of the analyses conducted to assess heterogeneity, pleiotropy, and sensitivity.

### 3.2 Causal impact of OCPC on the risk of systemic inflammation factors

**FIGURE 5:**
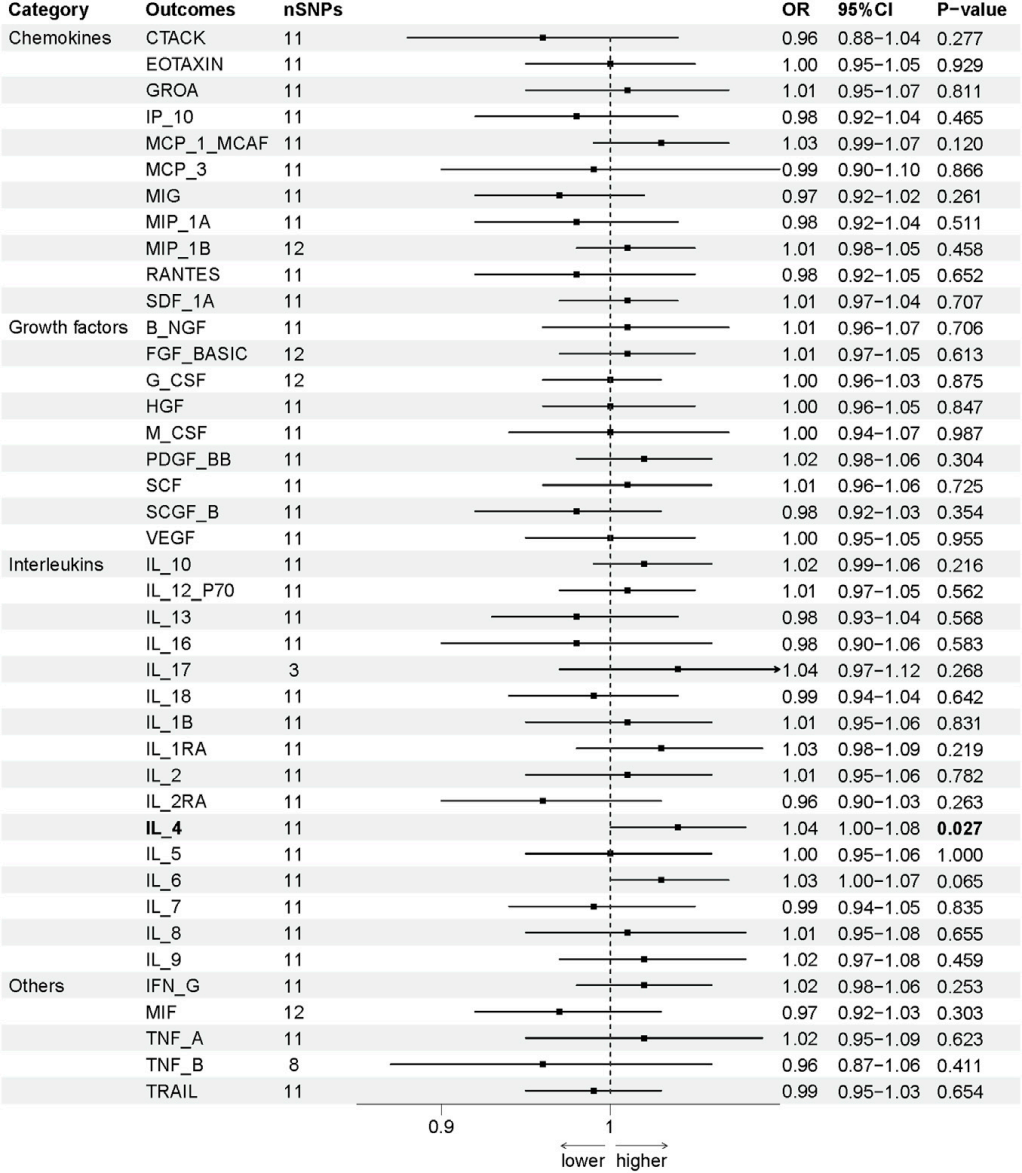
The influence of OCPC gene estimation on systemic inflammatory regulators (SNPs reaching *p* < 5 × 10^−6^) can lead to causal consequences. The connection between OCPC and levels of circulating cytokines can be predominantly ascertained through a two-sample Mendelian randomization (MR) analysis using the IVW technique. The estimated range of the 95% confidence interval (CI) reflects the odds ratio of circulating cytokines for each 1-SD rise in genetically anticipated OCPC levels. A detailed overview of cytokines can be found in Table 1.

**FIGURE 6:**
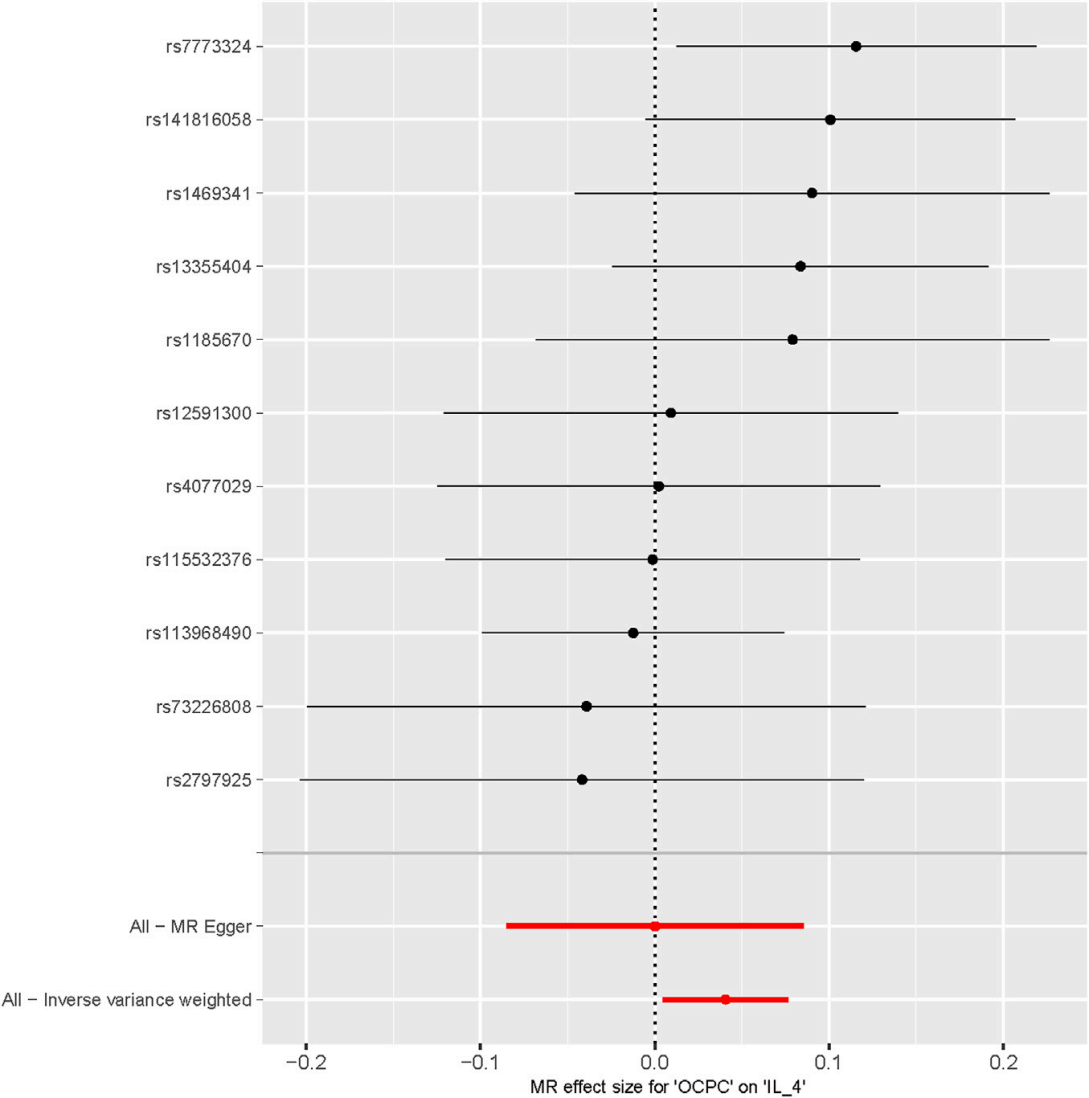
Forest plot of OCPC-related SNPs and IL-4 risk (The effect estimates of OCPC on IL-4 analyzed through MR are represented on the *X*-axis. Each SNP site’s MR effect values are represented on the *Y*-axis).

**FIGURE 7:**
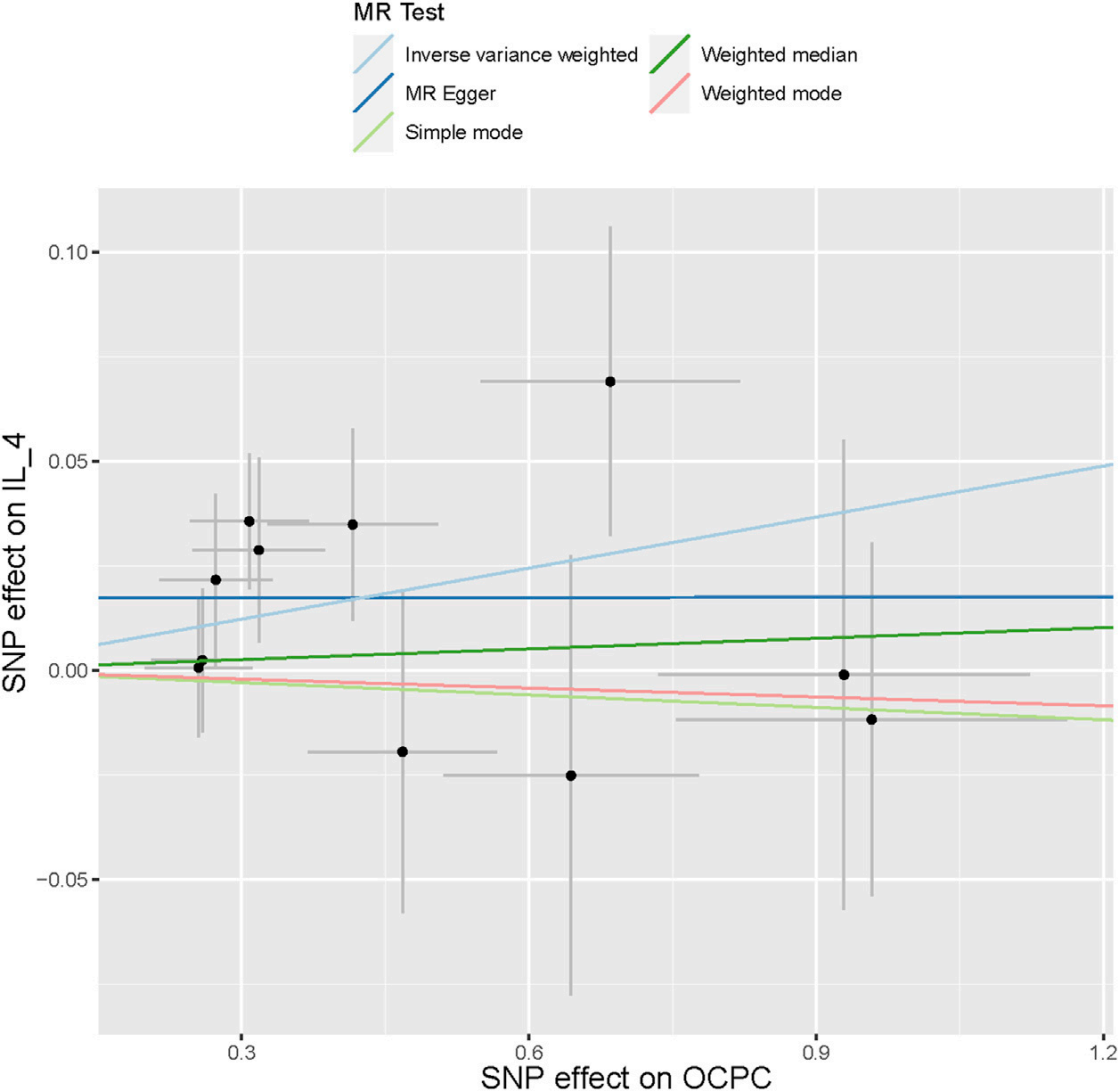
The scatter plot in this passage shows the genetic associations between SNPs related to OCPC and IL-4 using different MR methods. The gray error bars represent the 95% confidence intervals of the effects. These scatter plots represent the associations between each allele and the risk of the outcome, plotted against the association with one standard deviation of exposure. The analysis was conducted using IVW, MR Egger, Weighted median, Simple mode, and Weighted mode. The slope of each line represents the estimated MR effect of each method.

To evaluate the reverse causal effects, we conducted a study in which we identified 12 SNPs that have a significant and independent association with OCPC. The association reached a significance level of *p* < 5 × 10–^6^. We used a varying number of SNPs for each cytokine due to the lack of certain SNPs to be used universally. Detailed information about the number of SNPs used for each cytokine can be found in Supplementary Table S6. A suggestive association was observed between genetic susceptibility to OCPC and increased levels of Interleukin-4 (IL-4) based on the IVW method (OR: 1.04, 95%CI: 1.00–1.08, *p* = 0.03). No other significant associations were found, except for IL-4 (Figure 5). Furthermore, significant results of the MR and sensitivity analysis of OCPC and cytokines are shown in Table 3. Figures 6, 7 present forest plots and scatter plots illustrating the causal impact of OCPC-associated SNPs on IL-4.

**TABLE 3:** We evaluated the effects of genetic prediction (OCPC) on the levels of circulating IL-4. To assess heterogeneity between individual SNP estimates, we utilized Cochran’s Q test. The MR-Egger intercept test was employed to investigate horizontal pleiotropy.

Cytokines	Methods	MR results					Heterogeneity test			Horizontal pleiotropy test		
							Cochran’s Q test			MR-egger intercept test		
		SNPs	*β*	SE	*P*	OR (95% CI)	Q	df	*P*	Intercept	SE	*P*
IL-4	IVW	11	0.041	0.018	0.027	1.042 (1.005–1.080)	9.090	10	0.524	0.017	0.017	0.332
	MR Egger	11	0.000	0.044	0.996	1.000 (0.918–1.099)	8.040	9	0.530			
	Weighted median	11	0.008	0.026	0.741	1.009 (0.959–1.060)						
	Simple mode	11	−0.010	0.048	0.842	0.990 (0.900–1.099)						
	Weighted mode	11	−0.007	0.045	0.879	0.993 (0.909–1.099)						

Abbreviations: SNP, single nucleotide polymorphism; *β*, effect size of SNP on exposure; SE, standard error; OR, odds ratio; CI, confidence interval; df, degrees of freedom; IVW, inverse variance weighted.

### 3.3 Other factors and MVMR

**FIGURE 8:**
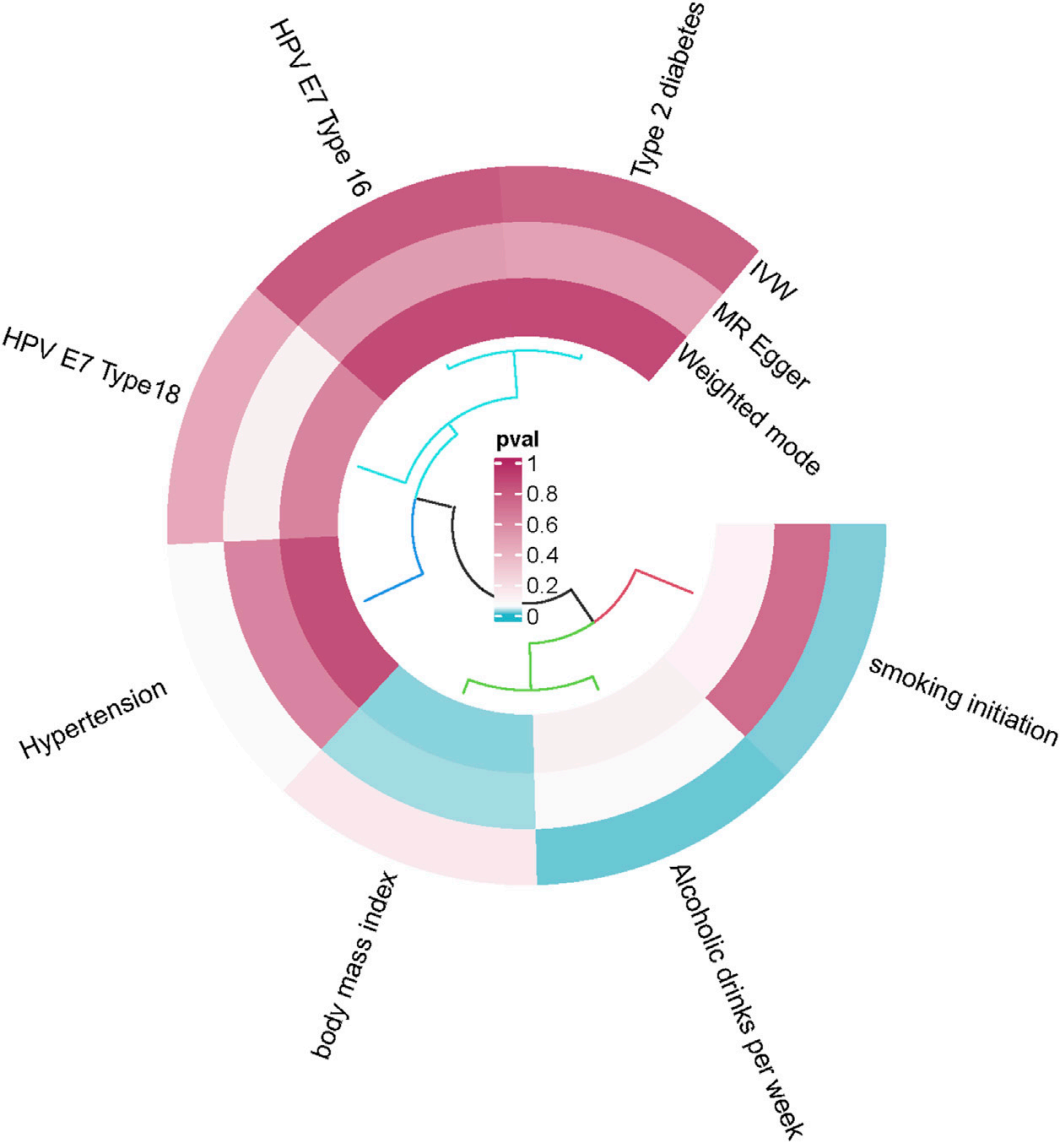
Causal effects of MIP1α/CCL3 and IL-7 on common risk factors for OCPC.

In univariate MR analysis, while examining the causal relationship between cytokines and OCPC, we also found that after removing SNPs associated with confounding factors, there were still some SNPs that were not only associated with OCPC but also strongly associated with other risk factors. Therefore, assessing the causal relationship between cytokines and common risk factors for OCPC is beneficial to identifying interfering factors that may mediate the association between cytokines and OCPC. We combined all SNPs related to MIP1α/CCL3 and IL-7 as cytokine instrumental variables to find the greatest genetic confounding. Preliminary results show a potential causal relationship between cytokines and common OCPC risk factors (including smoking, drinking, body mass index, and hypertension) (Figure 8, Supplementary Table S7). To control for pleiotropic pathways, when we further applied the MVMR model, the results showed that cytokines still have a potential causal effect on OCPC (MIP1α/CCL3, OR: 1.002, 95% CI: 0.919–1.092, *p* = 0.044; IL-7, OR: 0.997, 95% CI: 0.994–0.999, *p* = 0.011) (Table 4).

**TABLE 4:** MVMR results for OCPC.

Outcome	Exposure	SNPs, n	Or (95% CI)	*p*-value
OCPC	MVMR based on MIP1α/CCL3			
	MIP1α/CCL3	14	1.002 (0.919–1.092)	0.044
	Body mass index	455	1.000 (0.862–1.161)	0.076
	Smoking initiation	21	1.002 (0.575–1.182)	0.085
	Alcoholic drinks per week	6	1.002 (0.575–1.746)	0.283
	Hypertension	0	NA	NA
	MVMR based on IL-7			
	IL-7	43	0.997 (0.994–0.999)	0.011
	Body mass index	129	1.001 (0.783–1.279)	0.125
	Smoking initiation	14	1.001 (0.330–3.034)	0.566
	Alcoholic drinks per week	2	1.002 (0.254–3.949)	0.700
	Hypertension	0	NA	NA

Abbreviations: MVMR, multivariable Mendelian randomization; n, number; SNP, single-nucleotide polymorphism; OCPC, oral cavity and pharyngeal cancer; OR, odds ratio; CI, confidence interval.

## 4 Discussion

Numerous observational studies have found that the levels of circulating cytokines are related to the occurrence of OCPC. However, observational studies may be subject to bias due to inadequate sample size and confounding factors, resulting in skewed results. Moreover, there is insufficient genetic evidence to support this relationship in this field. Therefore, we utilized the latest GWAS data and adopted a systematic analysis approach to explore the causal effects of 41 different cytokines on OCPC. Unlike traditional MR studies, we not only focused on single cytokines but also performed bidirectional MR analysis, which can simultaneously test whether two factors have a causal relationship and what the causal direction is. In this way, we can identify the cytokines before or after the disease pathway. In addition, we also used various MR methods to enhance the robustness of the study and minimize the interference of pleiotropic effects. The objective of this study was to systematically assess the potential causal relationship between 41 circulating cytokines and OCPC risk using bidirectional MR analysis.

The tumor microenvironment and the malignant properties of tumor cells are crucial factors in shaping the biological behavior of tumors, directly driving their growth, invasion, and metastasis (Hanahan and Coussens, 2012). Systemic cytokines are a group of molecules that play a broad role in controlling inflammation throughout the body. These cytokines maintain the balance between pro- and anti-inflammatory processes in the TME, thereby ensuring that the immune system functions effectively during infection, injury, or disease while avoiding excessive damage to body tissues (Zhang et al., 2020; Ozga et al., 2021; Bhat et al., 2022). Accumulating evidence indicates that chemokines may exert pro-tumor effects in different types of cancer (Soria and Ben-Baruch, 2008; Levina et al., 2009; Hwang et al., 2012). Our findings show a positive association between high levels of CCL3 and an elevated risk of OCPC. CCL3, a chemokine that belongs to the CC chemokine subfamily, is synthesized by monocytes/macrophages, lymphocytes, neutrophils, as well as various immune cells including eosinophils, mast cells, fibroblasts, and dendritic cells. It is also called as macrophage inflammatory protein-1α (MIP-1α) (Hanahan and Coussens, 2012). CCL3 plays a pivotal role in recruiting inflammatory cells under both homeostatic and pathological conditions. CCL3 may contribute to cancer progression by stimulating leukocyte accumulation, angiogenesis, and tumorigenesis. OCPC cells and tissues exhibit overexpression of CCL3, which correlates with poorer survival rates among OCPC patients (Silva et al., 2007; da Silva et al., 2017). CCL3 can stimulate cancer cell growth and migration (da Silva et al., 2017; Hsu et al., 2013), whereas blocking CCL3 can suppress tumor growth, and angiogenesis, and increase cell sensitivity to therapeutic drugs (Liao et al., 2016; Kim et al., 2017). These mechanisms could elucidate the role of CCL3 in driving the pathophysiology of OCPC.

Research has shown that inflammation in specific organs can affect the risk of cancer, and inflammatory factors also exist in the tumor microenvironment to alter the proliferation, survival, and metastasis of malignant cells. An association between OCPC and inflammatory cytokines, wound-healing genes, growth factors, and cell cycle genes has been reported (Perez-Sayans et al., 2009; Mantovani, 2010). Interestingly, our study suggests that an increase of 1 standard deviation (SD) in the predicted levels of the IL-7 gene is related to a decreased risk of OCPC. IL-7, an indispensable cytokine for the adaptive immune system, is primarily secreted by the bone marrow, thymus, and lymph nodes. IL-7 plays a crucial role in lymphocyte development and survival, contributing to the maintenance of immune self-stability in the body (Fry and Mackall, 2002; Jiang et al., 2005; Hong et al., 2012; Barata et al., 2019). This effect may be due to the potential role of active inflammatory responses in decreasing the incidence of OCPC. Due to the powerful biological effects of IL-7, specifically, its role in promoting T cell longevity, growth, replication, and preservation of memory, various scientific groups have used IL-7 as a molecular enhancer to improve the immune response generated by cancer vaccines and maintain long-lasting memory reactions against cancer (Zhao et al., 2022). In conclusion, our study suggests a possible relationship between IL-7 and inflammatory tumors in OCPC, which may have some clinical significance for exploring the pathogenesis of OCPC and reducing its incidence.

We also explored potential mediators in the cytokines-OCPC pathway. Previous MR studies have shown that MIP1α/CCL3 and IL-7 are associated with OCPC risk. In observational studies, smoking, drinking, body mass index, type 2 diabetes, hypertension, and HPV16/18 infection have been suggested as risk factors for the development of OCPC. To account for the above potential mediating factors, the MVMR model was further applied to examine the possibility of the observed confounders introducing horizontal pleiotropy. However, MVMR analysis showed that OCPC was no longer significantly associated with these factors at the conventional 5% level. Therefore, it seems unlikely that these factors play a substantial role in the pathway of inflammatory exposure. Notably, MIP1α/CCL3 and IL-7 still have estimated causal effects on OCPC even if mediating factors are excluded.

Bidirectional MR analysis during the OCPC stage suggested that OCPC is potentially associated with alterations in IL-4 levels in blood, despite the limited available evidence on their correlation. Hypotheses propose that elevated levels of IL-4 might signify the presence of analogous immune-suppressing mechanisms within the microenvironment of OCPC. These mechanisms potentially facilitate the expansion of tumors and evasion of immune vigilance. Previous studies have reported significant IL-4 expression in the tumor microenvironment (Setrerrahmane and Xu, 2017; Gao et al., 2021). Inhibiting it can alter inflammation and improve the tumor’s response to immunotherapy (Ito et al., 2017). To sum up, the complex interactions between the immune system and the tumor are reflected by the varying IL-4 levels in the blood of patients with OCPC. These results highlight the importance of immune regulation and the imbalance of cytokines in the development of OCPC. Therefore, additional research is necessary to understand the exact mechanisms behind these changes in cytokines and how they impact the occurrence, advancement, and treatment strategies for OCPC.

This study is remarkable for its novel use of large-scale GWAS data to investigate multiple exposure factors related to OCPC risk, which enhances the stability and accuracy of estimating the effects. Second, the bidirectional MR design is employed to mitigate confounding factors and eliminate reverse causality. Third, the MVMR model was further applied to examine the possibility of introducing horizontal pleiotropy by common confounders of OCPC. Finally, this study comprehensively investigates the association between OCPC and 41 circulating cytokines, making it the most extensive MR study to date on this topic. They may provide oncologists and therapists with new perspectives and implications for designing more personalized and effective treatments for patients who may develop or suffer from OCPC. For example, host genotype could be exploited to enable more precise diagnosis and treatment by investigating inflammatory exposure factors in clinical patients. The use of monoclonal antibodies to reduce the concentration of certain inflammatory factors or exogenous supplementation of specific inflammatory factors can also be studied to prevent or treat OCPC. While the MR design offers advantages, this study also has limitations. First, although GWAS statistical data of European ancestry were used to mitigate population bias, the generalizability of our findings across different populations remains uncertain. Additional GWAS with larger sample sizes are necessary to validate and update the findings of this study. Moreover, the results of these studies may be confounded by various factors, including the production and interplay of cytokines. Despite conducting a MVMR analysis, it seems that the estimates of time-varying exposure may not accurately reflect the causal effects within a specific time period. When the exposure only affects the outcome at a few distinct time points and the risk factors in the MVMR analysis are the exposure values at these specific time points, it is possible to obtain a reliable estimation of the causal effect during these time points. However, if these time points are not correctly identified, estimates obtained from ambiguous models will be incorrect. This can mislead any inferences made about the magnitude, existence, or direction of the causal effects. Finally, it is important to note that all research findings require validation in clinical and basic research. As a result, caution should be exercised in interpreting potential causal relationships, and further investigation of potential physiopathological mechanisms is needed.

## 5 Conclusion

Our comprehensive MR analysis revealed potential causal relationships between 41 circulating cytokines and OCPC, providing new insights into their interactions. The following conclusions were drawn: MIP1α/CCL3 and IL-7 may be potential factors driving OCPC. And susceptibility to OCPC may also increase IL-4 levels in prognosis. However, due to the limitations of our study, including the relatively small number of OCPC cases and the use of only a single ancestry population, our findings should be validated in a larger cohort and the exact underlying biological mechanisms require further investigation.

## Data Availability

The datasets presented in this study can be found in online repositories. The names of the repository/repositories and accession number(s) can be found in the article/Supplementary Material.
