# Supplementary material for: Genetically predicted circulating levels of cytokines and the risk of oral cavity and pharyngeal cancer: a bidirectional mendelian-randomization study
Source: Front Genet. 2024 Jan 11;14:1321484. doi: 10.3389/fgene.2023.1321484 (PMC10808506; doi:10.3389/fgene.2023.1321484)
Supplement: Supplementary file 2 [file Image1.pdf]

Supplementary Material

Supplementary Figures

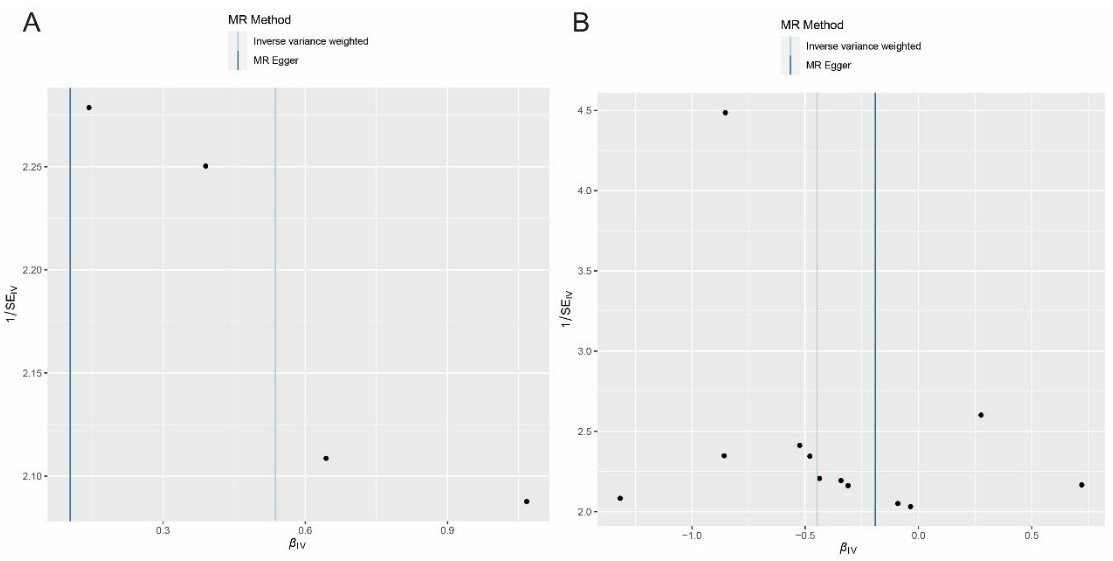

Supplementary Figure 1. Funnel plots for Mendelian Randomization analyses of the causal effect of MIP1α/ CCL3, IL-7 on OCPC.

A, Funnel plot for MR analyses of the causal effect of MIP1α/ CCL3 on OCPC. B, Funnel plot for MR analyses of the causal effect of IL-7 on OCPC. IVW and MR Egger methods were used to detect the heterogeneity of SNP. The funnel plots showed general symmetry, suggesting little evidence of heterogeneity. MR, Mendelian Randomization; IVW, Inverse variance weighted; SNP, single-nucleotide polymorphism.

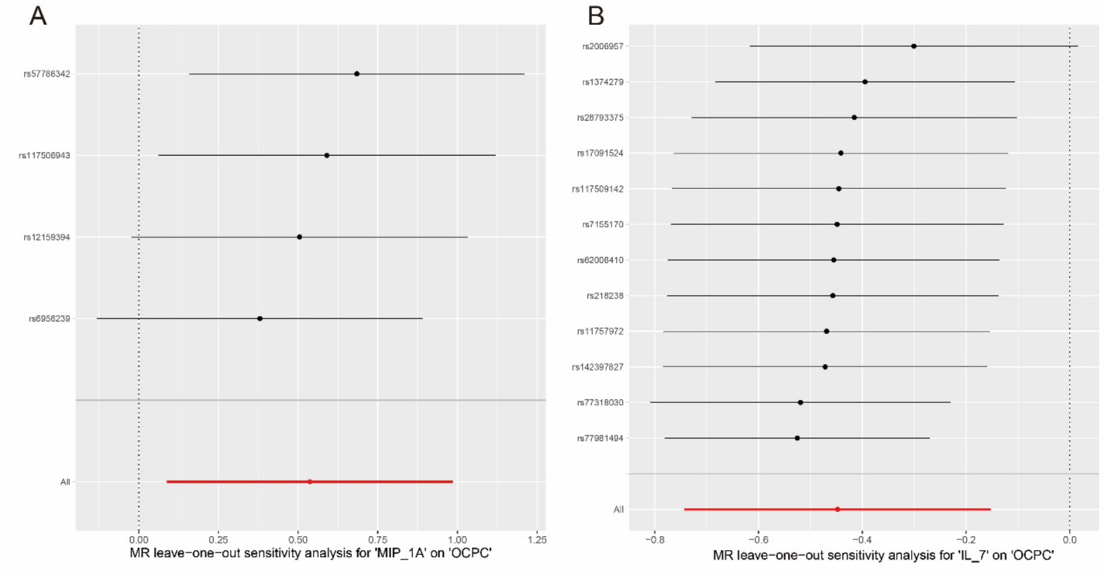

Supplementary Figure 2. Leave-one-out sensitivity based on IVW model for MIP1α/ CCL3, IL-7 on OCPC.

A, Leave-one-out sensitivity based on IVW model for MIP1α/ CCL3 on OCPC. B, Leave-one-out sensitivity based on IVW model for IL-7on OCPC. The overall estimate (red horizontal line) wasnot affected by the removal of a single variable (black horizontal line). There was no evidence of obvious heterogeneity, indicating that no specific SNP alone accounted for the association between MIP1α/IL-7 and OCPC. The results suggested that there was no individual SNP with a strong influence on the overall effect. SNP, single-nucleotide polymorphism; IVW, Inverse variance weighted.

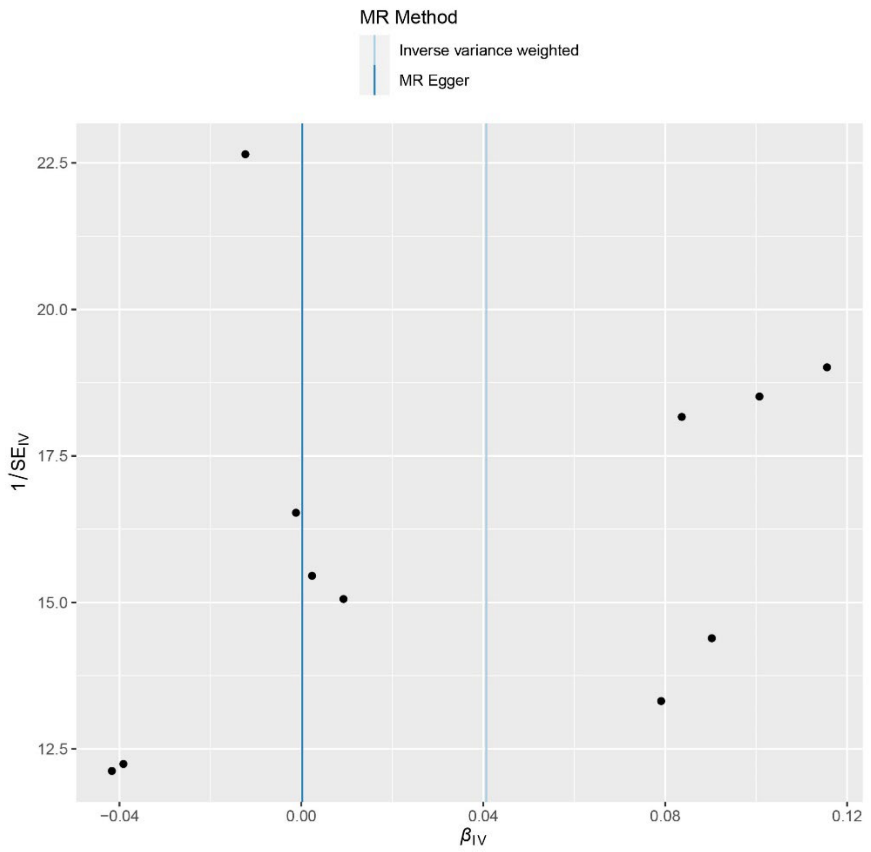

Supplementary Figure 3. Funnel plots for Mendelian Randomization analyses of the causal effect of OCPC on IL-4.

IVW and MR Egger methods were used to detect the heterogeneity of SNP. The funnel plots showed general symmetry, suggesting little evidence of heterogeneity. MR, Mendelian Randomization; IVW, Inverse variance weighted; SNP, single-nucleotide polymorphism.

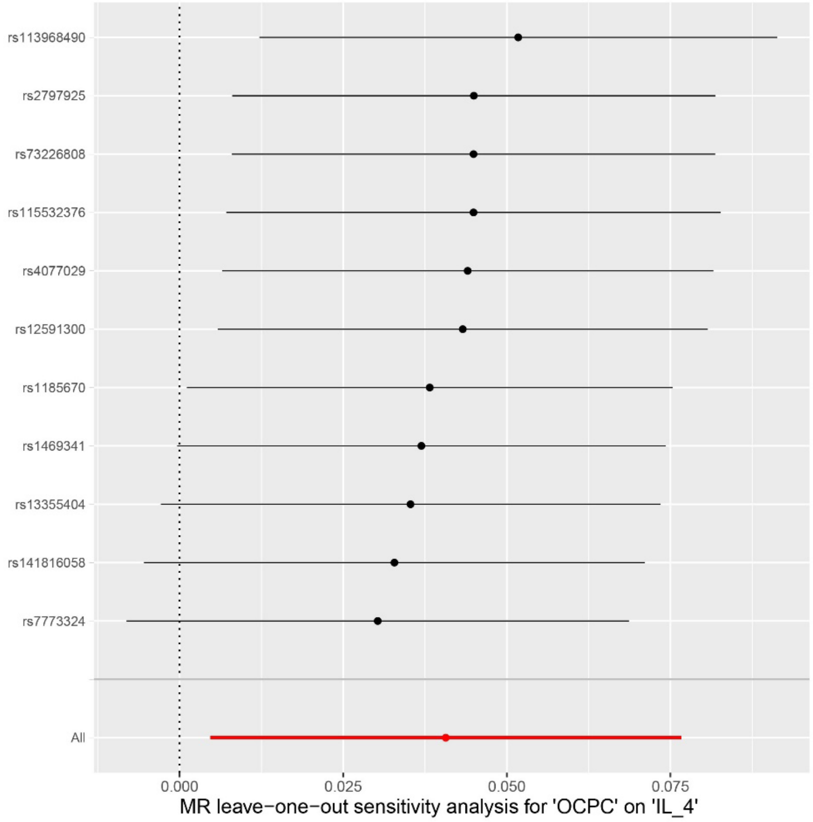

Supplementary Figure 4. Leave-one-out sensitivity based on IVW model for OCPC on IL-4.

The overall estimate (red horizontal line) wasnot affected by the removal of a single variable (black horizontal line). There was no evidence of obvious heterogeneity, indicating that no specific SNP alone accounted for the association between IL-4 and OCPC. The results suggested that there was no individual SNP with a strong influence on the overall effect. SNP, single-nucleotide polymorphism; IVW, Inverse variance weighted.
